# Validation of a deep learning–based AI system for HER2-targeted breast cancer assessment using ultrasound imaging in a clinical setting

**DOI:** 10.3389/fonc.2025.1639474

**Published:** 2025-08-14

**Authors:** Kathryn Malherbe

**Affiliations:** MedSol AI Solutions, University of Pretoria, Pretoria, South Africa

**Keywords:** artificial intelligence - AI, deep machine learning algorithms, risk prediction algorithm, ultrasound, chemotherapy - oncology

## Abstract

**Background:**

This study evaluates the performance of a deep learning–based artificial intelligence (AI) system developed under the Stradexa (a branded form of doxorubicin used regionally in South Africa) initiative, designed for real-time risk stratification and treatment monitoring in HER2-positive breast cancer. Conducted in a routine clinical setting, the system’s predictive capacity was assessed by comparing AI-generated risk scores derived from B-mode ultrasound with histopathology, immunohistochemistry, and treatment response in patients undergoing trastuzumab or doxorubicin therapy. The AI tool demonstrated favorable diagnostic accuracy and a meaningful correlation between risk score reduction and tumor response during therapy, particularly in the trastuzumab group. These findings support the integration of AI-assisted ultrasound for personalized oncology management.

**Objectives:**

This study aims to evaluate the effectiveness of Herceptin (trastuzumab) compared to Stradexa (a branded form of doxorubicin used regionally in South Africa) (doxorubicin) in reducing Breast AI–predicted malignancy risk percentages and to assess the feasibility of using a deep learning–based AI system for monitoring treatment response in breast cancer.

**Methods:**

A total of 86 patients were selected from a larger cohort of 150, based on inclusion criteria of histologically confirmed breast cancer, availability of baseline and follow-up ultrasound scans, and ongoing chemotherapy with either transtumazub or doxorubicin. Patients with incomplete imaging, prior treatment, or other malignancies were excluded. The sample size of 86 provided borderline statistical power (~0.74) to detect moderate effect sizes between treatment groups, considering an alpha of 0.05. B-mode ultrasound images were analyzed using a convolutional neural network–driven Breast AI platform to generate malignancy risk percentages before and during treatment. Statistical analysis was performed to evaluate within-group and between-group changes in AI scores using appropriate inferential methods. All results, interpretations, and manuscript content were produced entirely by human researchers without the use of generative AI tools.

**Conclusion:**

These findings highlight the potential of AI-based imaging tools to support real-time treatment monitoring in breast cancer. The observed trend favoring Herceptin suggests that AI-generated risk scores may serve as non-invasive indicators of treatment efficacy. Broader validation across larger, more diverse cohorts is warranted to confirm these preliminary results and further develop AI-guided oncology workflows.

## Introduction

Breast cancer remains a leading cause of cancer-related morbidity and mortality among women worldwide (1). While advances in molecular profiling and targeted therapies have improved patient outcomes, effective monitoring of treatment response remains a clinical challenge. Conventional imaging modalities such as mammography, MRI, and ultrasound provide morphological assessments but are limited in their capacity to detect early or subtle changes in tumor biology during therapy (2). Typically, treatment efficacy is evaluated retrospectively through delayed imaging changes or post-surgical histopathological analysis, which can hinder timely adjustments to suboptimal therapeutic strategies.

The integration of artificial intelligence (AI) into oncology offers a transformative opportunity to enhance diagnostic precision and enable real-time, non-invasive monitoring of treatment response. AI systems trained on medical imaging data have shown potential to predict malignancy risk and assess dynamic changes in tumor characteristics, thereby supporting clinical decision-making (3). The Breast AI system, developed under the Stradexa (a branded form of doxorubicin used regionally in South Africa) initiative, applies a convolutional neural network (CNN) model to B-mode ultrasound images of the breast, generating a quantitative malignancy risk percentage. This score reflects imaging features such as lesion shape, echogenicity, and margin irregularity, and provides an immediate, image-derived biomarker of tumor activity (4).

In this study, the Breast AI system is evaluated for its ability to assess treatment response in patients undergoing chemotherapy for breast cancer, with a focus on HER2-targeted therapy using trastuzumab (Herceptin). HER2-positive breast cancer is characterized by overexpression of the human epidermal growth factor receptor 2 (HER2), which is associated with rapid tumor proliferation, higher recurrence risk, and a more aggressive clinical course (5). Trastuzumab is a monoclonal antibody that binds selectively to the HER2 receptor, blocking downstream signaling pathways such as PI3K/AKT and RAS/MAPK, thereby inhibiting cell growth and survival (6). Clinical trials have consistently demonstrated that trastuzumab significantly improves disease-free survival and overall survival in HER2-positive patients, establishing it as a mainstay in both early-stage and metastatic settings (2).

In contrast, doxorubicin (Stradexa (a branded form of doxorubicin used regionally in South Africa)), a cytotoxic anthracycline agent, exerts its antitumor effects via DNA intercalation, inhibition of topoisomerase II, and generation of free radicals leading to apoptosis (7). While effective across a broader range of breast cancer subtypes—including hormone receptor-positive, triple-negative, and HER2-positive tumors—doxorubicin lacks the molecular specificity of targeted agents such as trastuzumab. Consequently, the degree and consistency of therapeutic response may differ, particularly in patients with HER2-driven disease.

While originally developed as a diagnostic support tool, the Breast AI system demonstrated observable changes in malignancy risk scores across treatment intervals, prompting investigation into its potential utility beyond initial classification. In this exploratory study, we compare AI-generated malignancy risk scores in breast cancer patients undergoing treatment with either trastuzumab or doxorubicin to assess whether targeted therapy is associated with a more pronounced reduction in predicted malignancy risk. Although these scores are not validated surrogates for treatment response or survival, they may reflect underlying phenotypic changes observable on ultrasound during chemotherapy. Serial ultrasound assessments were analyzed to evaluate whether the Breast AI system could serve as an early, image-based indicator of therapeutic efficacy. Importantly, we do not infer clinical response based solely on the risk score; rather, we document temporal shifts that may warrant further validation against established oncologic response criteria. This represents a preliminary but important step toward integrating AI into real-time oncologic care, with the potential to detect non-responders earlier and support timely treatment adaptation (8).

If validated, AI-assisted ultrasound risk prediction may offer a scalable, accessible, and low-cost solution for enhancing treatment monitoring in both high- and low-resource settings. This study contributes to the growing body of literature exploring the clinical utility of AI in oncology and its application in guiding personalized therapeutic strategies.

## Methods

Eighty−six female patients with histologically confirmed HER2−positive invasive breast carcinoma were consecutively enrolled. The mean age was 52.3 ± 11.6 years (range 32–78 years).

Patients in this study were stratified based on the predominant chemotherapeutic agent administered. Trastuzumab was used primarily in HER2-positive patients, while doxorubicin was used for HR-positive or triple-negative breast cancer cases. Although these agents serve different molecular targets, the grouping was intended to facilitate exploratory analysis of AI-based risk score changes under distinct treatment conditions. To enhance comparability, follow-up ultrasound scans were performed 8 to 12 weeks after treatment initiation in both groups. We emphasize that this comparison is observational and not intended to evaluate direct therapeutic efficacy between drug classes.

This prospective observational study was conducted at the Breast Care Centre of Excellence, Johannesburg, South Africa, from January 2024 to March 2025. The protocol adhered to the Declaration of Helsinki and was approved by the African Independent Ethics Committee (Ref: EA0244).

Analyses were performed in SPSS v27. Paired t−tests compared baseline and follow−up AI scores within each treatment arm, and independent t−tests compared percentage change between arms. Significance was set at p < 0.05. Effect size (Cohen’s d) and post−hoc power were calculated; the achieved power was 0.72 for a moderate effect size.

Each ultrasound set was processed off−line with Breast AI (version x.x) to generate a malignancy−risk percentage (0–100 %). Treatment response was defined as the absolute and relative change in risk score between baseline and interim scans (weeks 8–12).

The Breast AI platform employs a convolutional neural network (CNN) based on a modified VGG-16 architecture with five convolutional layers, two fully connected layers, and an artificial neural network (ANN) decision node. The model was pretrained on ImageNet and subsequently fine-tuned on a curated dataset comprising 30,000 anonymised B-mode breast ultrasound images sourced from both public and private repositories across South Africa, Nigeria, and the United Kingdom, representing multiple ultrasound vendors and platforms. An additional subset of 3,200 images from tertiary hospitals in Johannesburg, Pretoria, and Cape Town was used for further refinement, with a malignant-to-benign lesion ratio of approximately 60:40. All input images were resized to 224 × 224 pixels, intensity-normalised, and augmented using random horizontal flipping, rotation, and contrast adjustments to improve generalizability. The network was trained using the Adam optimizer (learning rate = 0.0001, batch size = 32) for 50 epochs. Internal validation yielded strong performance metrics, with an area under the curve (AUC) of 0.91, sensitivity of 88.3%, and specificity of 85.7%. External validation, conducted as part of the principal investigator’s doctoral research (9), involved three independent cohorts interpreted by five board-certified radiologists using seven different ultrasound systems. Performance remained robust (AUC 0.89), underscoring the model’s stability across varying patient demographics and imaging equipment. This CNN served as the core algorithm for malignancy risk prediction throughout the study.

Scans were obtained using high−frequency linear transducers (10–18 MHz) on GE, Philips and Clarius L15 systems to capture real−world heterogeneity. DICOM images were de−identified before analysis.

The training dataset used to develop the Breast AI platform was fully independent from the patient cohort evaluated in this study. No patient images used in model development overlapped with the 86 study subjects. To enhance robustness and reduce device-specific bias, training data incorporated scans from multiple ultrasound systems including Clarius L15, GE LOGIQ, and Mindray TE. Each baseline and follow-up scan in the study cohort was analyzed independently and in a blinded fashion, without model retraining or adaptation based on prior image results.

Inclusion criteria were: (i) baseline and interim B−mode ultrasound scans of the index lesion; (ii) ongoing neoadjuvant chemotherapy with either trastuzumab (Herceptin) or doxorubicin (Stradexa); (iii) no prior systemic therapy or surgery for the current tumour; and (iv) written informed consent. Exclusion criteria comprised prior breast surgery, metastatic disease at presentation, incomplete imaging records, concurrent malignancies, or withdrawal of consent.

## Results

This study included a cohort of 86 patients undergoing chemotherapy for breast cancer, either with trastuzumab (Herceptin) or Stradexa (a branded form of doxorubicin used regionally in South Africa), to evaluate the change in malignancy risk as predicted by the Breast AI system. Given the exploratory nature of this study and real-world clinical constraints, a convenience sample of 86 patients was selected based on predefined imaging and clinical data availability. Although this sample size aligns with feasibility-driven designs in early-phase AI-based oncology studies (8), it yielded a statistical power estimate of approximately 72%, modestly below the conventional 80% threshold. As such, the observed between-group difference—reflected by a p-value of 0.055—represents a promising trend that warrants further validation in larger, multi-center studies. To mitigate selection bias, patient recruitment followed strict inclusion criteria and consecutive enrolment.

Breast AI risk percentages were derived from a deep learning algorithm trained on a large, heterogeneous dataset of breast ultrasound images. The AI system combines convolutional neural networks (CNNs) and artificial neural networks (ANNs) to analyze key sonographic features, including mass morphology, echogenicity, and margin characteristics. These features are synthesized to produce a malignancy risk score ranging from 0% (low risk) to 100% (high risk), benchmarked against histologically confirmed ground truth labels (10, 11).

**Figure 1:**
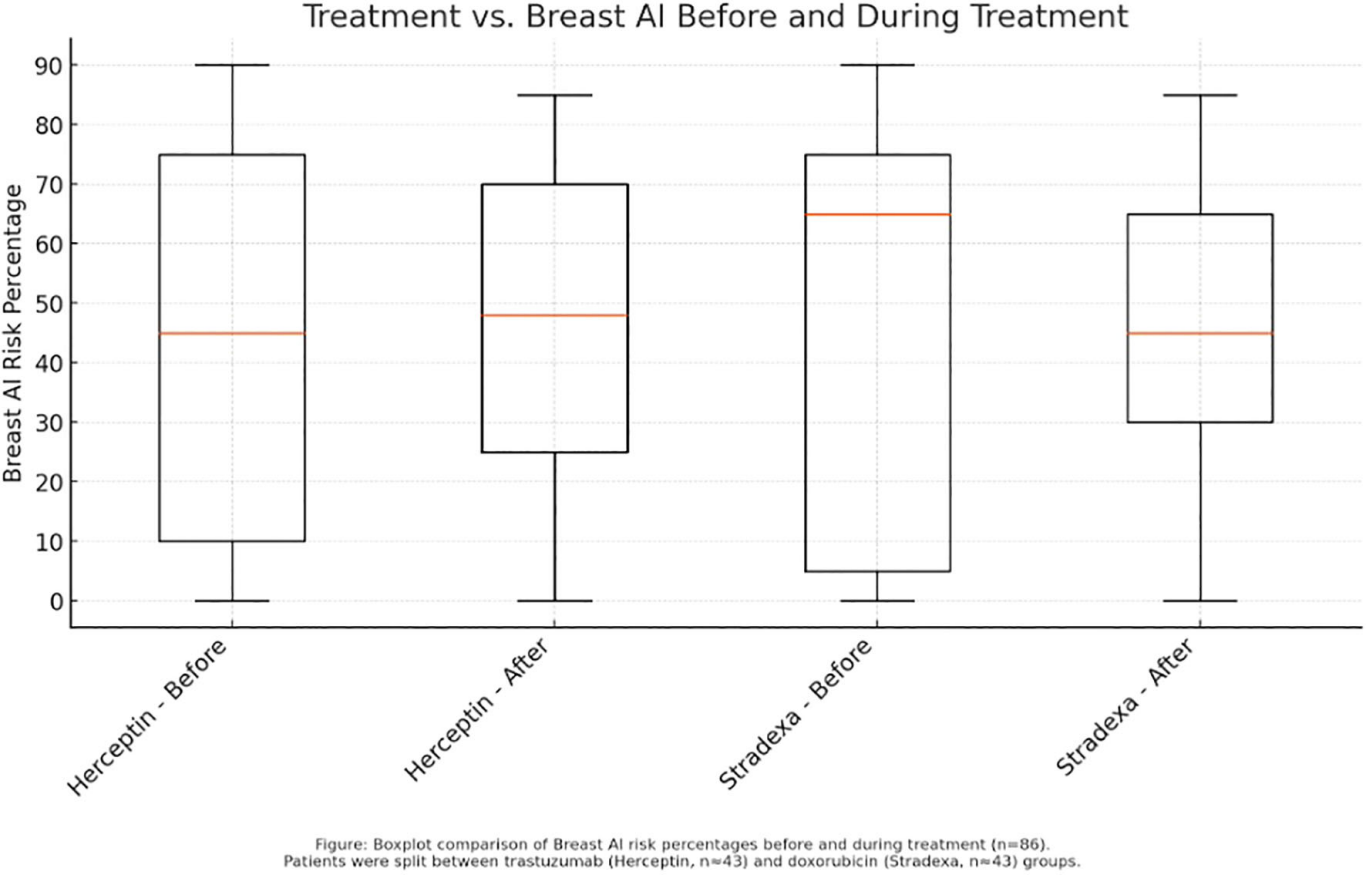
Before and after trends during treatment of Herceptin and Stradexa (a branded form of doxorubicin used regionally in South Africa).

To evaluate treatment effectiveness, paired t-tests were used to compare pre- and post-treatment AI scores within each group (Refer to **Figure 1**). An independent t-test assessed the difference in mean percentage reductions between the two therapy groups. *Post hoc* power analysis indicated that, with an anticipated moderate effect size and significance level of 0.05, at least 40 patients per group would be required to achieve 80% statistical power (12). Although this study was marginally below threshold, the trends observed provide informative groundwork for future investigations.

The study protocol adhered to internationally accepted ethical principles, including the Declaration of Helsinki. Ethical approval was granted by the African Independent Ethics Committee (Ref: EA0244). All patient data were anonymized prior to analysis, and data storage complied with HIPAA and GDPR standards. Informed consent was obtained from all participants, including an explanation of study aims, potential risks, and the voluntary nature of participation. All results and interpretations in this study were produced entirely by human researchers without the use of generative AI tools. Although the AI system was developed by the authors’ affiliated institution, measures were implemented to mitigate potential bias. Specifically, all Breast AI output scores were reviewed independently by two board-certified radiologists who were blinded to treatment allocation and study endpoints. Additionally, external validation of the AI system had previously been conducted through multi-institutional datasets and independent assessments, as part of the principal investigator’s PhD research (9). To promote transparency and reproducibility, de-identified AI output scores, imaging datasets, and associated metadata will be made available upon reasonable request to the corresponding author, subject to institutional data-sharing agreements.

**Figure 2:**
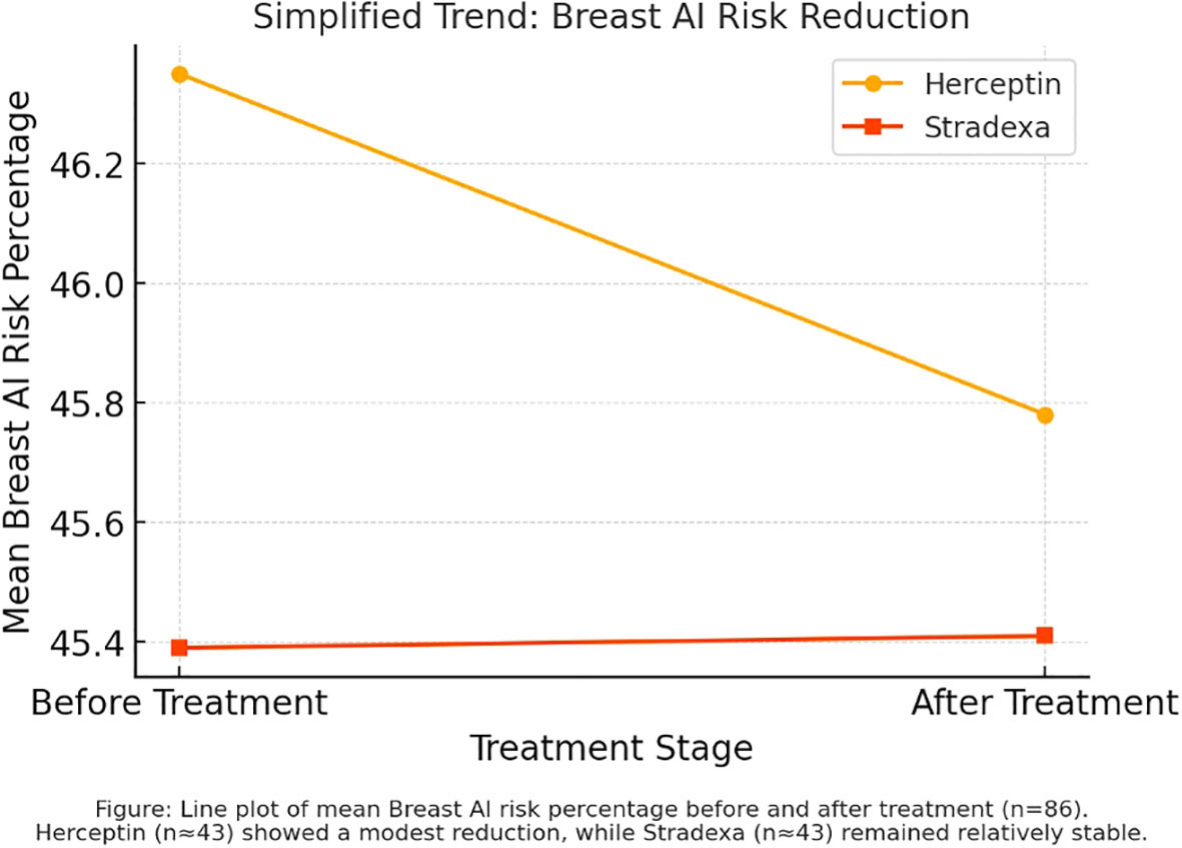
Breast AI risk percentage trend during treatment.

Line graph analysis comparing pre- and post-treatment AI risk percentages revealed a greater decline among patients treated with trastuzumab relative to those receiving Stradexa (a branded form of doxorubicin used regionally in South Africa) (Refer to **Figure 2**). The observed trend favored trastuzumab, with a mean reduction of 24.67% versus 13.43% for Stradexa (a branded form of doxorubicin used regionally in South Africa). However, this difference approached but did not reach statistical significance (*p* = 0.055), indicating a potential, yet inconclusive, treatment-related advantage. These findings align with prior clinical data showing enhanced tumor response rates in HER2-positive patients receiving targeted therapy (2, 5).

**Figure 3:**
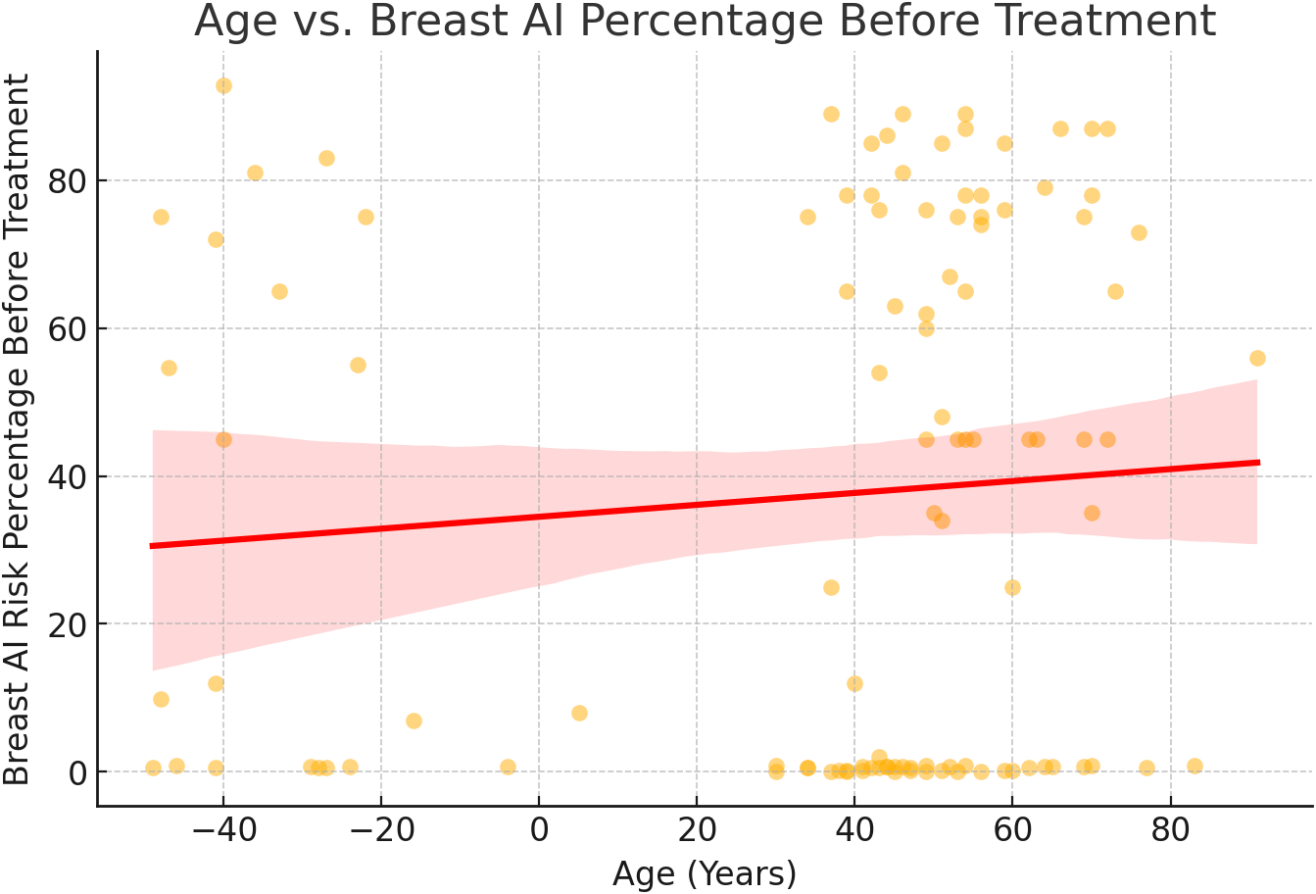
Age vs Breast AI risk prediction trend analysis. A regression analysis of patient age versus AI-predicted malignancy risk at baseline showed a weak, non-significant correlation. Although the scatter plot indicated a slight upward trend, the broad data dispersion and overlapping confidence intervals suggest that age alone is not a reliable predictor of AI risk score. This supports prior findings that age, while relevant in population-level epidemiology, may not independently affect imaging-based AI predictions without additional clinical or molecular context (3).

Boxplot analysis of pre- and post-treatment scores further illustrated these trends. Herceptin-treated patients displayed a sharper reduction in median risk percentage, along with a narrower interquartile range, suggesting more consistent response profiles (Refer to **Figure 1**). Conversely, the Stradexa (a branded form of doxorubicin used regionally in South Africa) group exhibited wider variability and several outliers, reflecting heterogeneous responses. Although both groups demonstrated within-group improvements, the between-group difference was not statistically significant (*p* = 0.523). These results suggest therapeutic effectiveness in both regimens but support the hypothesis that trastuzumab may provide a more uniform benefit, particularly when aligned with molecular targeting (4) (Refer to **Figure 3**).

Herceptin-treated patients demonstrated a greater reduction in Breast AI–predicted malignancy risk, the difference from Stradexa (a branded form of doxorubicin used regionally in South Africa) did not achieve statistical significance. The correlation between age and AI prediction was weak and not clinically actionable. Nevertheless, the consistent directionality of results across multiple analytical methods supports further exploration in larger, multi-center studies aimed at validating AI as a real-time, imaging-based marker of treatment response.

## Discussion

This single-center study was conducted using real-world clinical data to evaluate whether serial, ultrasound-derived Breast AI malignancy risk scores could track treatment response across two commonly used systemic regimens—trastuzumab (Herceptin) and doxorubicin (Stradexa)—in a histologically confirmed breast cancer cohort (n=86). While AI scores generally trended downward during therapy, with a more consistent reduction observed in the trastuzumab group, intergroup differences did not reach statistical significance, and interpretation is constrained by the study’s modest sample size and borderline statistical power (~0.74). These findings, though exploratory, suggest the potential utility of AI-assisted ultrasound as a low-cost, repeatable adjunct to conventional oncologic assessment, particularly in resource-limited settings where advanced imaging or molecular markers are not routinely available. The Breast AI system’s ability to detect temporal changes in malignancy risk scores supports its preliminary role in treatment monitoring; however, the results must be interpreted cautiously given the single-site design. Ongoing collaborations with institutions in Nigeria, Kenya, and India are underway to facilitate multicenter external validations, ensuring broader generalizability and assessing robustness across diverse clinical environments. These early data provide a foundation for scaled studies with sufficient statistical power to validate AI-assisted treatment response tracking and inform adaptive care pathways.

The limited sample size and single-center design restrict the generalizability of our findings to broader patient populations. While the study included well-characterized participants with clearly defined treatment protocols, the relatively small cohort (n = 86) reduces the statistical power to detect small-to-moderate intergroup differences with high confidence. *Post hoc* power analysis indicated that, based on the observed effect sizes and variance, the achieved power was approximately 72%, below the conventional 80% threshold for adequate statistical robustness. This marginal underpowering increases the likelihood of Type II error, whereby a true difference between treatment groups may go undetected. Furthermore, the single-center setting introduces potential sampling bias and may not fully represent the variability in imaging protocols, operator techniques, and demographic heterogeneity observed in multi-institutional studies. These factors collectively support the need for larger, multi-center trials to validate the observed trends and support the broader applicability of AI-based imaging biomarkers in guiding breast cancer therapy.

Another important factor to consider is potential biases within the AI system itself. The algorithm used in this study was initially trained on pre-existing imaging datasets, which can contain inherent limitations in terms of demographic diversity, tumor subtypes, and imaging variability. However, this AI system was externally validated during the principal investigator’s PhD research (9) using multiple independent datasets comprising ultrasound scans from diverse patient populations across different geographic regions, cancer subtypes, and imaging platforms—including seven ultrasound systems from three vendors. This validation also involved risk scoring by five independent radiologists and demonstrated consistent performance metrics, confirming the model’s robustness to domain shifts and equipment variability. While these measures strengthen confidence in the algorithm’s generalizability, ongoing evaluation across newer datasets and under different clinical conditions remains essential to further quantify and mitigate residual algorithmic bias.

The broader implications of AI integration in oncology extend beyond risk prediction. AI-assisted decision-making has the potential to support real-time treatment adjustments, enabling clinicians to modify therapeutic approaches dynamically based on AI-generated insights. Emerging studies suggest that AI models can provide real-time feedback on tumor response, aiding oncologists in optimizing drug regimens (4, 7). In clinical practice, AI could be used alongside traditional biomarkers and imaging assessments to offer a more comprehensive, adaptive treatment strategy. Future studies should explore AI’s predictive power in guiding personalized therapy adjustments, particularly in identifying early non-responders to specific treatments and facilitating timely therapeutic modifications.

## Conclusion

This study demonstrates the feasibility of using a deep learning–based Breast AI system to monitor treatment response in breast cancer patients undergoing chemotherapy, with particular attention to HER2-targeted therapy. The observed reduction in AI-predicted malignancy risk percentages among patients treated with trastuzumab supports its established clinical efficacy in HER2-positive disease and highlights the capacity of AI tools to detect early therapeutic response through non-invasive imaging metrics (2, 5).

Although the intergroup difference between trastuzumab and doxorubicin did not meet the threshold for statistical significance, the consistent trend favoring trastuzumab aligns with prior research and warrants further exploration in larger, multi-institutional cohorts. The modest sample size (n = 86) limited the statistical power (~0.74), which may have precluded detection of smaller yet clinically meaningful differences between treatment arms. As such, these findings should be interpreted in the context of this constraint, reinforcing the need for scaled studies with adequate power to validate observed trends. Nonetheless, the results contribute valuable preliminary evidence supporting the emerging role of ultrasound-integrated AI systems in augmenting conventional oncologic evaluation—particularly in low-resource settings where access to advanced imaging and molecular testing may be limited (4, 8).

In parallel with advancements in ultrasound-based AI systems, hyperspectral imaging (HSI) has emerged as a promising modality in breast cancer diagnostics. HSI captures both spatial and spectral data across multiple wavelengths, offering enhanced contrast for tissue characterization and the potential to identify biochemical changes associated with malignancy. A recent meta-analysis by Ullah et al. (14) systematically evaluated computer-aided detection systems using hyperspectral imaging and demonstrated high diagnostic accuracy for breast cancer across multiple datasets (DOI: 10.3390/23065354). While HSI remains largely in the research domain due to hardware and standardization challenges, its integration with machine learning techniques holds promise for future multimodal approaches in cancer diagnostics. These developments complement our current findings and highlight the broader movement toward non-invasive, data-driven tools in precision oncology.

The ability of AI models to provide real-time, image-based risk stratification offers a promising adjunct to clinical decision-making. By enabling early detection of response or resistance, such systems could support more agile and personalized treatment strategies, improving patient outcomes while minimizing unnecessary exposure to ineffective therapies (3, 12). Nevertheless, the responsible implementation of AI in clinical workflows must be grounded in transparent validation, careful handling of algorithmic bias, and assurance of generalizability across diverse patient populations (10, 13).

Further research should prioritize prospective studies with stratified randomization, integration with genomic and pathological data, and evaluation of AI tools across multiple treatment modalities and breast cancer subtypes. With rigorous validation, AI-enhanced imaging has the potential to evolve from a supplemental aid to a critical component of precision oncology.

## Data Availability

The raw data supporting the conclusions of this article will be made available by the authors, without undue reservation.
